# Visualizing knowledge evolution trends and research hotspots of artificial intelligence in colorectal cancer: A bibliometric analysis

**DOI:** 10.3389/fonc.2022.925924

**Published:** 2022-11-28

**Authors:** Guangwei Liu, Jun Zhao, Guangye Tian, Shuai Li, Yun Lu

**Affiliations:** ^1^ Department of Gastrointestinal Surgery, The Affiliated Hospital of Qingdao University, Qingdao, Shandong, China; ^2^ Department of Pharmacy, The Affiliated Hospital of Qingdao University, Qingdao, Shandong, China; ^3^ School of Control Science and Engineering, Shandong University, Jinan, Shandong, China; ^4^ State Key Laboratory of Virtual Reality Technology and Systems, Beihang University, Beijing, China; ^5^ Shandong Key Laboratory of Digital Medicine and Computer Assisted Surgery, Qingdao University, Qingdao, Shandong, China

**Keywords:** bibliometric analysis, artificial intelligence, network visualization, colorectal cancer, deep learning

## Abstract

**Background:**

In recent years, the rapid development of artificial intelligence (AI) technology has created a new diagnostic and therapeutic opportunity for colorectal cancer (CRC). Numerous academic and clinical studies have demonstrated that high-level auxiliary diagnosis and treatment systems based on AI technology can significantly improve the readability of medical data, objectively provide a reliable and comprehensive reference for physicians, reduce the experience gap between physicians, and aid physicians in making more accurate diagnosis decisions. In this study, we used bibliometric techniques to visually analyze the literature about AI in the CRC field and summarize the current situation and research hotspots in this field.

**Methods:**

The relevant literature on AI in the field of CRC research was obtained from the Web of Science Core Collection (WoSCC) database. The software CiteSpace was utilized to analyze the number of papers, countries, institutions, authors, journals, cited literature, and keywords of the included literature and generate a visual knowledge map. The present study aims to evaluate the origin, current hotspots, and research trends of AI in CRC using bibliometric analysis.

**Results:**

As of March 2022, 64 nations/regions, 230 institutions, 245 journals, and 300 authors had published 562 AI-related articles in the field of CRC. Since 2016, each year has seen an exponential increase. China and the United States were the largest contributors, with the largest number of beneficial research institutions and the closest collaboration relationship. The World Journal of Gastroenterology is this field’s most widely published journal. Diagnosis and treatment research, gene and immunology research, intestinal polyp research, tumor grading research, gastrointestinal endoscopy research, and prognosis research comprised the six topics derived from high-frequency keyword cluster analysis.

**Conclusion:**

In recent years, field research has been a popular topic of discussion. The results of our bibliometric analysis allow us to comprehend better the current situation and trend of this research field, and the quantitative data indicators can serve as a guide for the research and application of global scholars.

## Introduction

Colorectal cancer (CRC) is one of the most prevalent malignant tumors. In 2020, there will be more than 1, 9 million new cases and 940,000 deaths worldwide, placing it third in terms of morbidity and second in terms of mortality. It is anticipated that the number of new cases of CRC will rise to 2.5 million by 2035, posing a grave threat to human life and health (1). In recent years, thanks to the support of clinical data, artificial intelligence (AI) has made significant advancements in the medical field and has been applied in various fields. Numerous intelligent, innovative information processing technologies have emerged in recent years (2). The application of AI in medicine consists primarily of two components. One is the virtual application form represented by “deep learning”, which relies on the continuous improvement of computer capabilities and the development of statistics through continuous learning and the accumulation of experience from data, as well as mathematical algorithms to improve machine learning. The other is the application form represented by “physical medicine”, which primarily consists of physical medical objects, medical equipment, and robots (3).

A large number of summary studies are also included in the published related studies, indicating that many researchers pay close attention to the progress and direction of AI research in the field of CRC. However, traditional literature retrieval and review research has a general subjective bias because it emphasizes the content and extracts representative papers from existing literature.

Bibliometrics based on big data and statistical analysis reduces to some extent the common subjective bias in the research progress of traditional literature retrieval and review forms, and the results presented *via* digitization and visualization are more objective and trustworthy. Moreover, using visual processing, we can quantitatively and qualitatively evaluate the research trends in the research field, reveal the most productive authors and institutions and the current research hotspots, and predict future research trends (4, 5). Presently, bibliometric analysis is widely used to study the development trend of numerous subjects and disciplines, and many fruitful efforts have also been made in AI (6–8).

The purpose of the reported bibliometric analysis is to create a knowledge map of AI in CRC research, which can systematically demonstrate the global academic community’s research status and development trend and offer literature data support and reference for formulating research strategies and directions.

## Methodology

### Data sources

The literature search was conducted using the Web of Science Core Collection (WoSCC) database. Two researchers compared their respective findings. This procedure was repeated twice, once by the author and once by the co-author. The search query was TS=((“artificial intelligence” OR “deep learning”) AND (“colorectal cancer” OR “colon cancer” OR “rectal cancer”)). To ensure that the internalized literature was representative, the document types were restricted to “article” and “review”, and the language was restricted to English. From 1985.1.1 to 2022.3.23, the publication timeframe was analyzed. Duplicate and non-representative items such as conference papers, news, and errata were removed.

### Data analysis

The records (complete records and cited references) retrieved from the WoSCC database in plain text file format were imported into CiteSpace 5.8 R1 software to discuss this study’s dynamic development and trend research. The time slicing parameters were set from January 1999 to March 2022 (the pertinent literature on the application of AI in CRC was first published in 1999). The time slicing parameters were set to 1 year. Using CiteSpace software, the country or region, author, and institution were analyzed. The journal double image overlay utilized the CiteSpace software’s Overlay Maps function. Meanwhile, VOSviewer and Carrot 2 software were employed for keyword co-occurrence analysis. The keyword emergence analysis used CiteSpace’s Burstiness function. Then, parameters were set for each node type (country, author, institution, and keywords) and visual analysis, the knowledge map of research, the co-occurrence, emergence, and clustering knowledge maps of keywords, as well as the time axis map and time zone map of keywords were generated.

## Results

### Research trends

From the WoSCC database, 745 AI-related CRC-related papers were retrieved. After excluding non-literature papers, the follow-up analysis included 562 of the remaining papers.

The number of papers published in various years reflected the researchers’ commitment to this field. In 1999, Anandk combined k-nearest neighbor (KNN) and Genetic Algorithms (GA) to create the first report in this field: an AI prognosis model for CRC patients (9).

**Figure 1:**
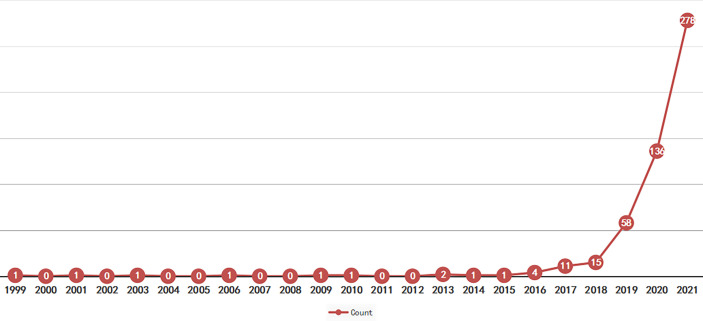
Publication trend with respect to time.

Since then, the number of papers published between 1999 and 2015 had been relatively low, with only ten papers published in 16 years and no relevant research results published for a considerable time. The research has increased exponentially since 2016, with an exponential trend line of y = 1.6573e^0.8601x^, R^2^ = 0.9849, reaching a peak of 278 in 2021. This increase results from the maturation of AI technology and the expansion of research. As of March 2022, 50 papers have been published in this field. The number of papers published in 2022 may be lower than in 2021, but it will still be higher than in 2021. It is hypothesized that AI applications will continue to receive a great deal of attention and will continue to be the focus of future research. **Figure 1** provides specific information.

### Contribution by country or region

**Figure 2:**
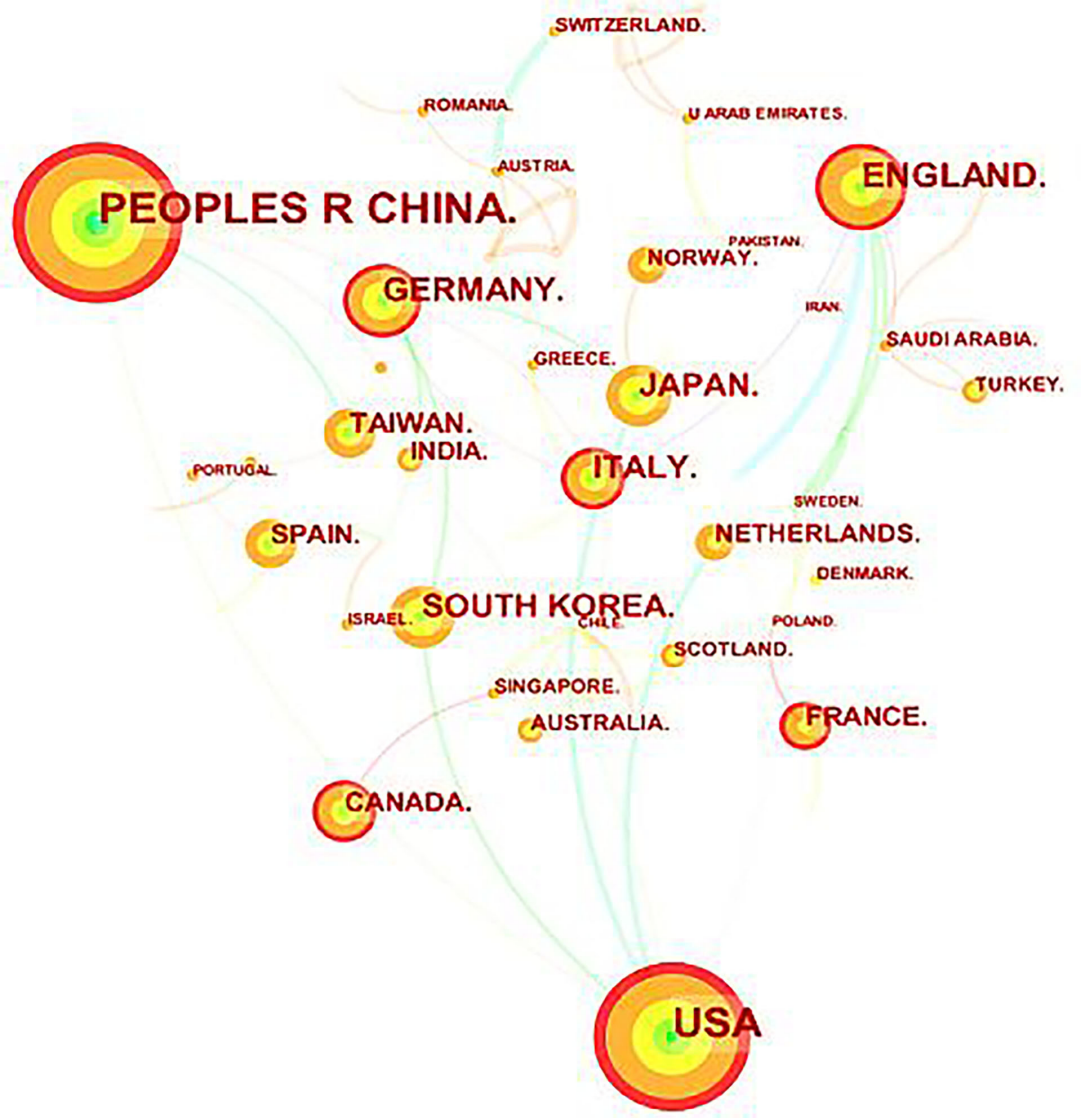
Co-countries analysis of global research.

**Figure 3:**
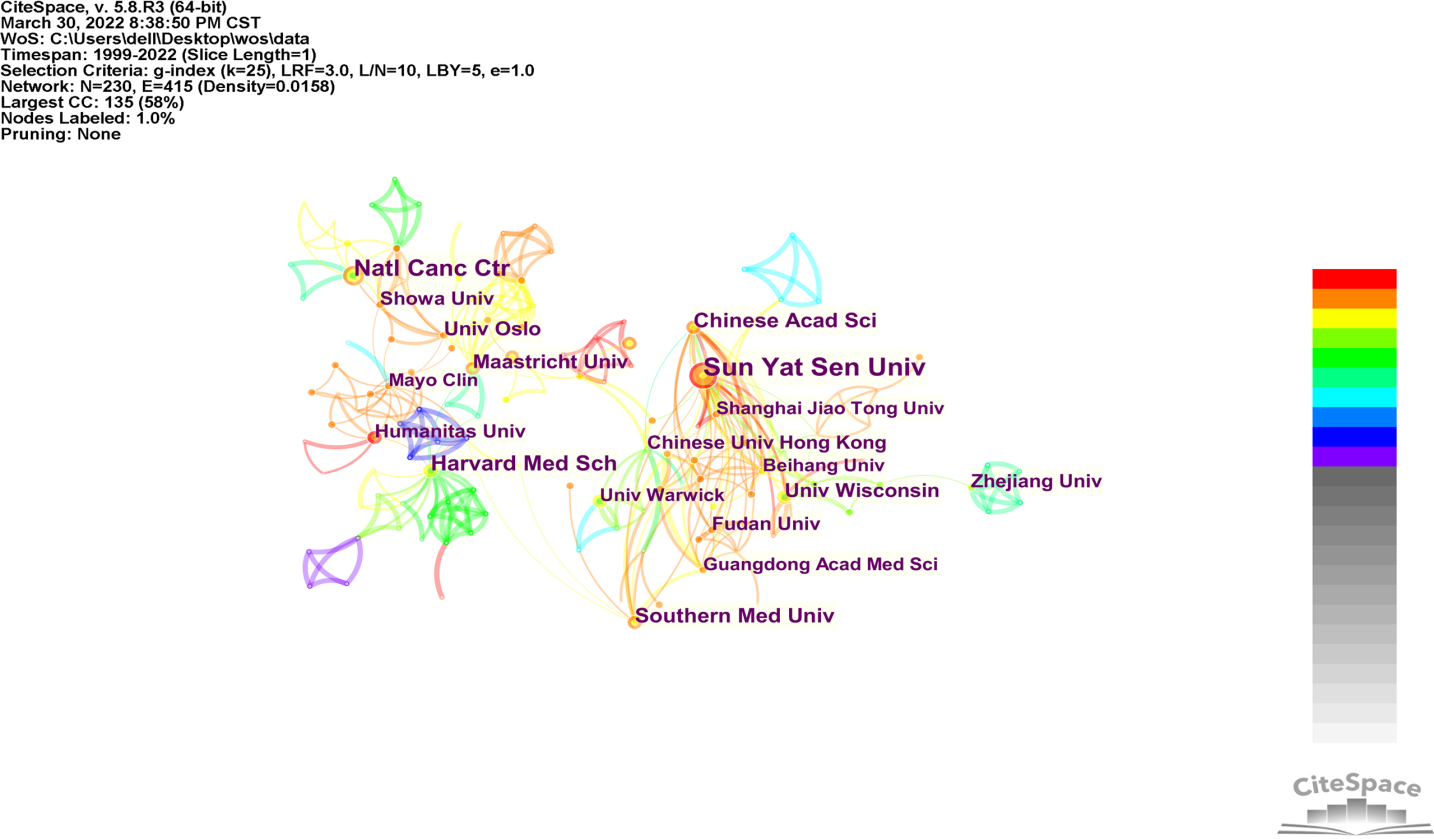
Co-institutions analysis of global research.

The papers originated from 64 countries and regions (**Figure 2**). Twenty-four countries or regions have published more than five papers (**Figure 3**). China (154), the United States (131), the United Kingdom (56), Germany (41), South Korea (40), Japan (40), Italy (38), Taiwan (23), Canada (21), and France (20) rounded out the top ten countries (**Table 1**). The number of documents issued by China and the United States (USA) was significantly higher than that of any other country, which reflects the academic level and standing of these two nations in this field and demonstrates that they play significant leadership roles in determining the direction of research in this field. In addition, although the number of papers published in the United Kingdom (UK) accounted for only 9.96% of all papers published, their citations were the highest (33.55), indicating that British scholars have published a large number of high-quality papers. Although China has been active in this field and has produced more achievements in recent years, there is still a gap in the quality and influence of research results compared to the UK and the USA, and the proportion of ground-breaking research results must be increased.

**Table 1:** Top 10 of most produtive countrie/region.

Number	Country/Regions	Number of publication	Percentage	Citation	Average citations per paper
1	China	154	27.40%	2265	14.71
2	USA	131	23.31%	3954	30.18
3	England	56	9.96%	1879	33.55
4	Germany	41	7.30%	656	16
5	South Korea	40	7.12%	312	7.8
6	Japan	40	7.12%	474	11.85
7	Italy	38	6.76%	308	8.11
8	Taiwan	23	4.09%	413	17.96
9	Canada	21	3.74%	81	3.86
10	France	20	3.56%	174	8.7

**Figure 4:**
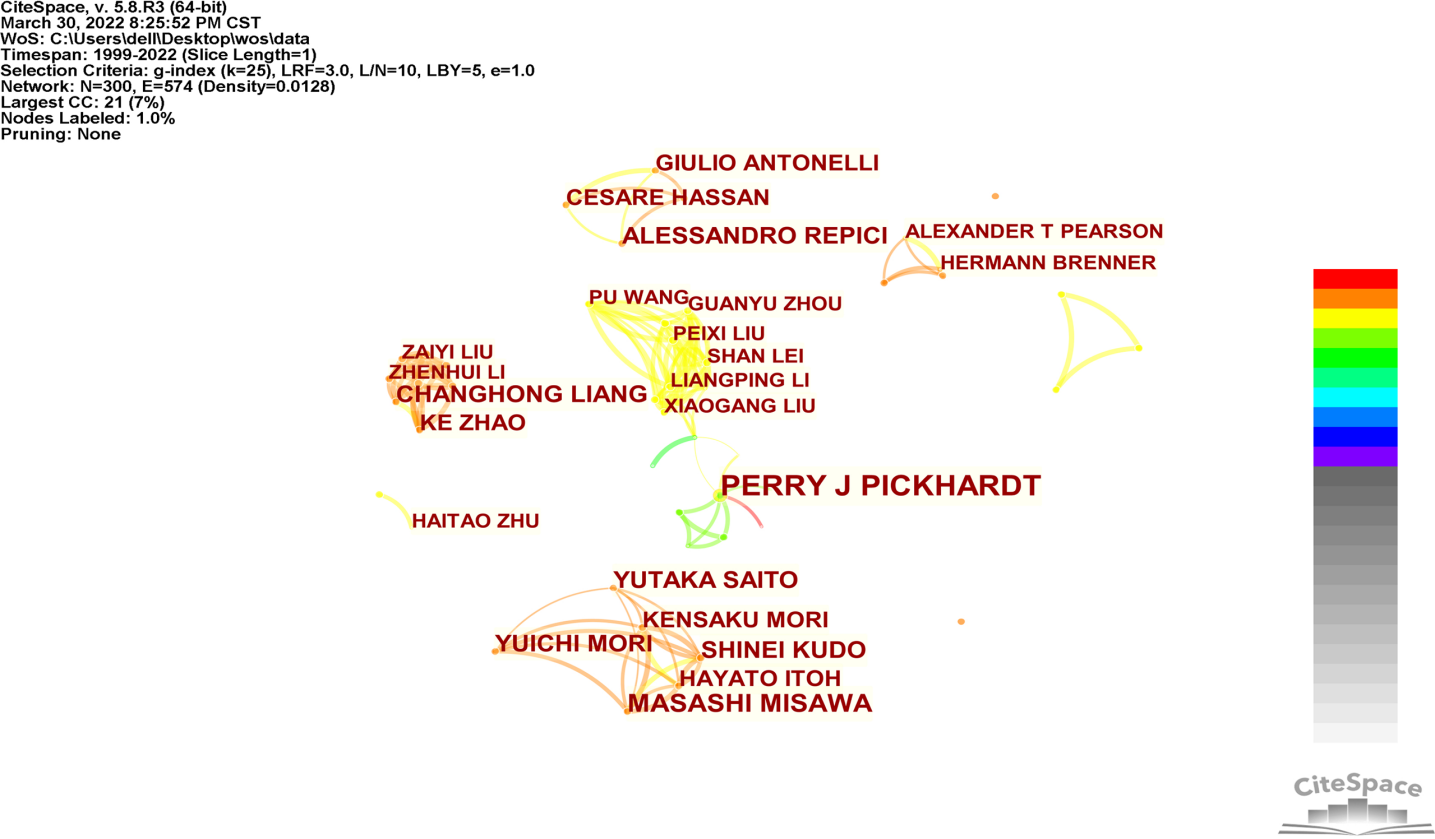
Co-authors anaysis of global research.

**Figure 2** depicts a visual network map of the source countries’ cooperation to comprehend better the cooperation relationship between various countries in this field. Different colors represent various countries or regions; the area of each color represents the amount of literature published in each country or region; the thickness of the connection indicates the cooperation frequency. **Figure 4** demonstrates that various countries have united and collaborated in this field, contributing to the breakthrough and innovation in this field. The USA and China, the countries with the most publications, have demonstrated a very close working relationship. In addition, the USA works closely with Italy, Germany, and the UK, whereas China works closely with Singapore and the UK.

### Contribution by institution

The main research visualization map (**Figure 3**) included 230 research institutions, 415 lines, and a network density of 0.0158. The greater the size of the dot, the greater the number of published papers. The closer the partnership, the stronger the connection. We discovered that Sun Yat Sen University (20, 3.56%), National Cancer Center (14, 2.49%), Harvard Medical School (12, 2.56%), Southern Medical University (10, 1.78%), and the Chinese Academy of Sciences (10, 1.78%) were the top five research institutions in terms of AI application. Concurrently, numerous research institutions have engaged in extensive cooperation in this field, forming two major research groups in China, Europe, and the USA. In contrast, China’s scientific research institutions predominately engage in domestic cooperation. **Table 2** lists the top 15 research institutions, seven of which are from China, three from the USA, one from the Netherlands, one from Norway, one from Italy, one from Japan, and one from the UK.

**Table 2:** Top 15 of most productive institution.

No	Institutions	Country	Count	Percentage
1	Sun Yat Sen University	China	20	3.56%
2	National Cancer Center	USA	14	2.49%
3	Harvard Medical School	USA	12	2.14%
4	Southern Medical University	China	10	1.78%
5	Chinese Academy of Sciences	China	10	1.78%
6	Maastricht University	Netherlands	9	1.60%
7	University of Wisconsin	USA	9	1.60%
8	University of Oslo	Norway	9	1.60%
9	Humanitas University	Italy	8	1.42%
10	Fudan University	China	8	1.42%
11	Zhejiang University	China	8	1.42%
12	The Chinese University of Hong Kong	China	8	1.42%
13	Showa University	Japan	8	1.42%
14	Beihang University	China	7	1.25%
15	The University of Warwick	England	7	1.25%

### Contribution of authors

The authors’ collaboration network (**Figure 4**) revealed that 300 researchers worldwide contributed to the research, involving 574 lines and a network density of 0.0128. Researchers collaborated to form several research groups with the same research focus and consistent membership. In addition, six of the twelve researchers who published more than five articles were Japanese, three were Italian, two were Chinese, and one was American. **Table 3** provides the results.

**Table 3:** Top 12 of most productive authors (Related studies reported＞5) .

No	Authors	Count	Country
1	Perry J Pickhardt	9	USA
2	Masashi Misawa	7	Japan
3	Changhong Liang	6	China
4	Shinei Kudo	6	Japan
5	Alessandro Repici	6	Italy
6	Yutaka Saito	6	Japan
7	Yuichi Mori	6	Japan
8	Hayato Itoh	5	Japan
9	Giulio Antonelli	5	Italy
10	Kensaku Mori	5	Japan
11	Cesare Hassan	5	Italy
12	Ke Zhao	5	China

### Journals upon publication

A total of 562 papers were published in 245 journals, with around 2.29 articles per journal and an impact factor of 6.906. The top ten journals (**Table 4**) published more than seven papers, and the number of papers published by these ten journals represented 22.60% (127/562) of the total papers. The World Journal of Metrology published the most results (21 articles), Metrology had the highest impact factor (22.682), and IEEE Transactions on Medical was the most cited journal (256.71). Moreover, most published journals belonged to Q1 and Q2 medical journals with high impact factors and academic influence, indicating that AI is more likely to be utilized in CRC.

**Table 4:** Top 10 of most productive journal.

RANK	Journal	Count	Percentage	IF	Citations	Average citations per paper
1	World Journal of GastroenterologyNTEROLOGY	21	3.76%	5.742(Q2)	44	2.10
2	Caners	20	3.58%	6.639(Q1)	157	7.85
3	**Scientific Reports**	18	3.22%	4.38(Q1)	748	41.56
4	Frontiers in Oncology	15	2.68%	6.244(Q2)	27	1.8
5	IEEE Access	13	2.33%	3.367(Q2)	49	3.76
6	Applied Sciences-Basel	10	1.79%	2.679(Q3)	61	6.10
7	Diagnostic	8	1.43%	3.706(Q2)	25	3.13
8	Sensors	8	1.43%	3.576(Q2)	28	3.50
9	Gastroenterology	7	1.25%	22.682(Q1)	687	98.14
10	IEEE Transactions on Medical Imaging	7	1.25%	10.048(Q1)	1797	256.71

Q1 and Q2 represent the influencing factors of journals rank the top 25% and >25%-50% in the given subject in 2019, respectively.

**Figure 5:**
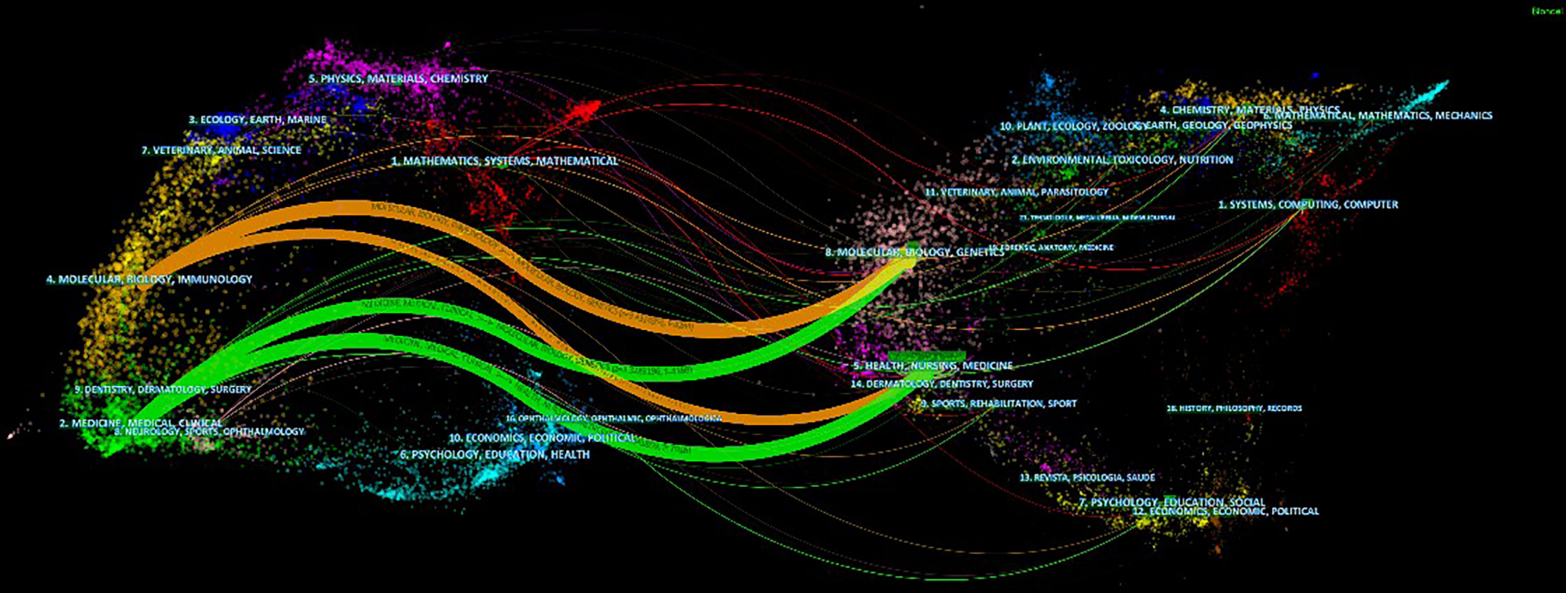
A dual-map overlay of journals related to AI on CRC.(Left) Citing journals. (Right) Cited journals. The color of the links distinguishes the discipline of the source.

**Figure 5** depicts a double map overlay of research topics between cited journals and CRC-cited journals. The labels on the map indicated the research topics that the journals covered. On the map, the citation journals are on the left, while the cited journals are on the right. The most published articles were “molecular biology, biology, and immunology” and “medicine and clinical”. In contrast, the most cited papers were published in “nursing, health, and medicine” and “molecular biology, biology, and genetics”. Moreover, the citation path indicated that it was published in “medicine, medical”. The research published in “clinical” was more likely to cite journals in “health, nursing, and medicine”.

### Keyword o-occurrence analysis

**Figure 6:**
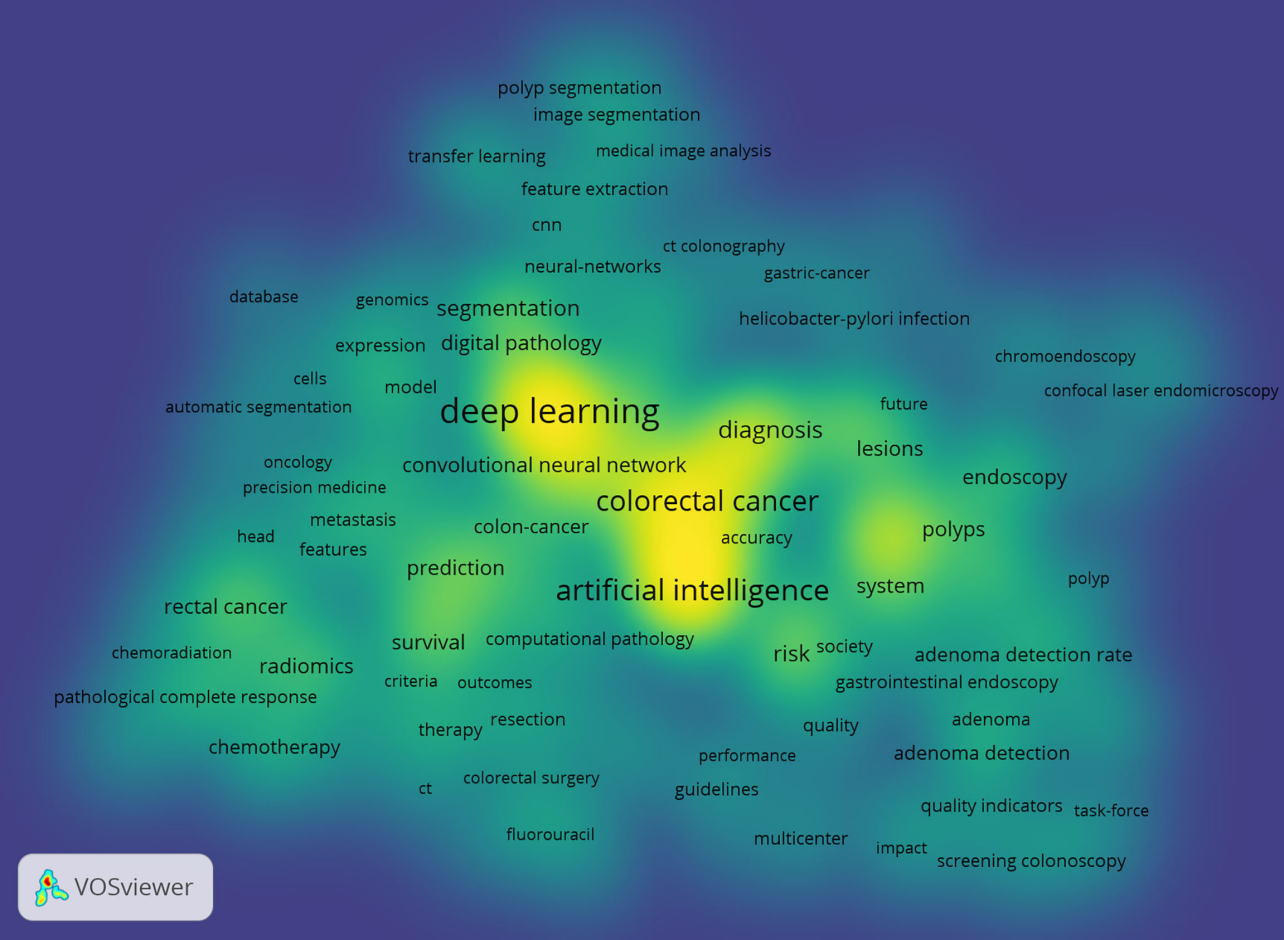
Density distribution of keywords clusters.

A total of 2,256 keywords were extracted from 562 articles, and the VOSviewer software was used to generate the visualization map. There were 183 keywords with at least five occurrences. **Figure 6** illustrates the visual density map.

**Figure 7:**
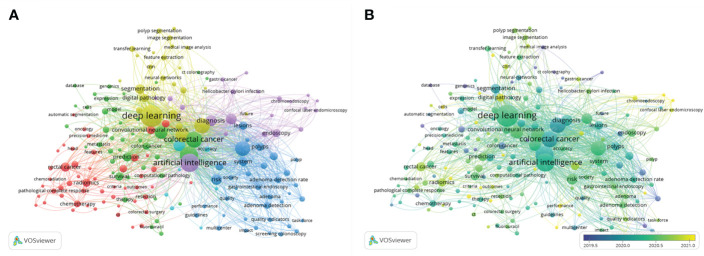
Network visualization **(A)** and overlay visualization map **(B)** of the keywords co-occurrence analysis by VOS viewer.

Based on the cluster analysis result, the keywords were divided into six clusters (**Figure 7A**): cluster 1 (red, diagnosis and treatment research, 50 keywords), cluster 2 (green, gene and immunology research, 43 keywords), cluster 3 (dark blue, intestinal polyp study, 36 keywords), cluster 4 (yellow, tumor grade study, 25 keywords), cluster 5 (purple, gastrointestinal endoscopy, 21 keywords), and cluster 6 (light blue, prognosis study, eight keywords). Cluster 1 was the largest cluster, with the algorithm, automatic segmentation, radiotherapy and chemotherapy, image analysis, and metastasis as the most critical aspects. The overlay visual map can color keywords differently based on the year they first appeared. The results are depicted in **Figure 7B**. In the time process, purple nodes represented earlier keywords, while yellow nodes represented the most recent. Recently, critical terms related to gastrointestinal endoscopy have emerged, indicating a new hotspot for research in this field.

**Figure 8:**
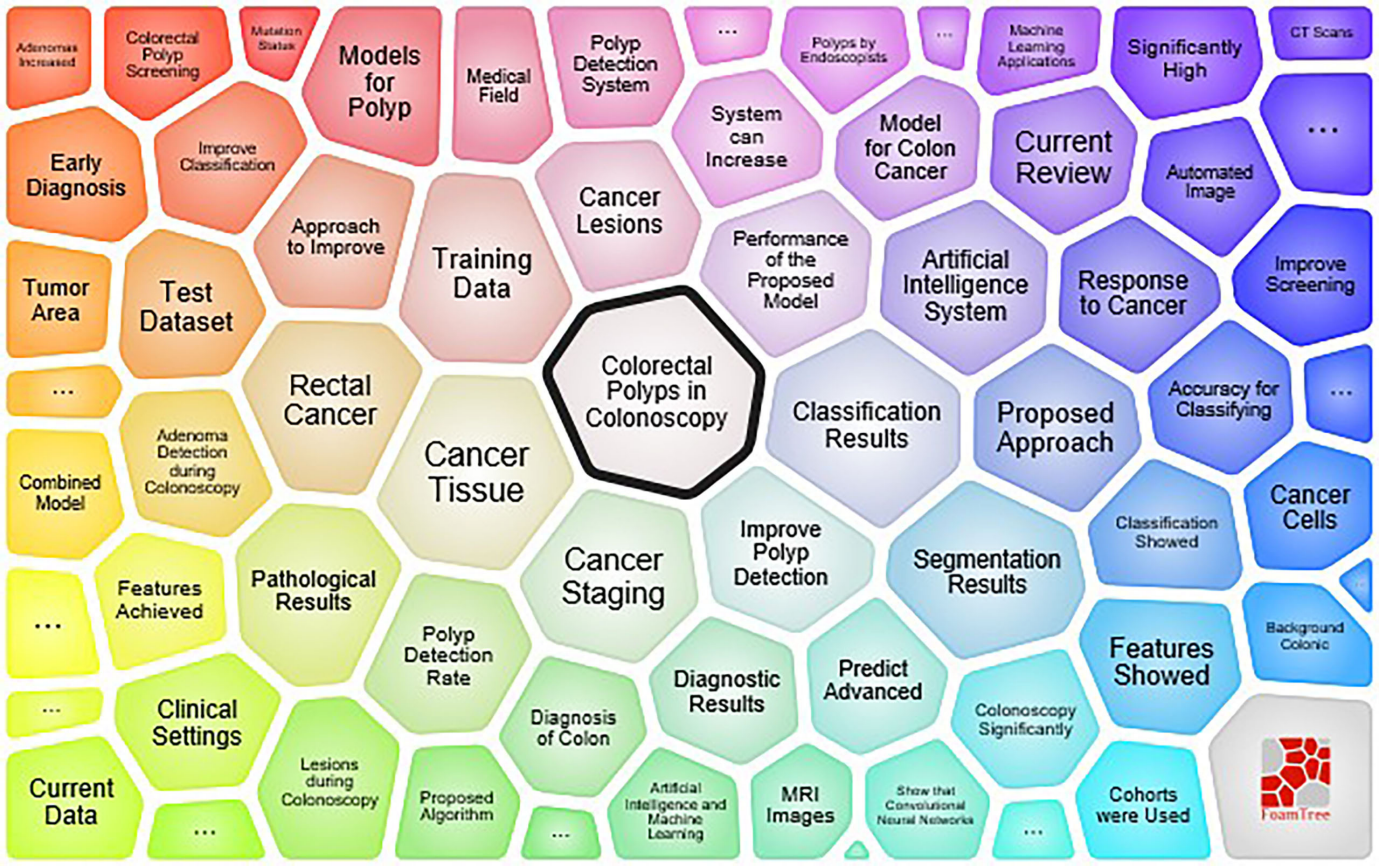
major topic survey based on the carrot system.

In addition, the key concepts were displayed using the foamtree function of the Carrot2 software, and the keyword with the highest occurrence rate was “colored polyps in colonoscopy”, confirming the conclusion that the application of AI in gastrointestinal endoscopy has garnered considerable attention. The remainder consisted of “classification results”, “cancer tissue” and “segmentation results”. **Figure 8** demonstrates the results.

**Figure 9:**
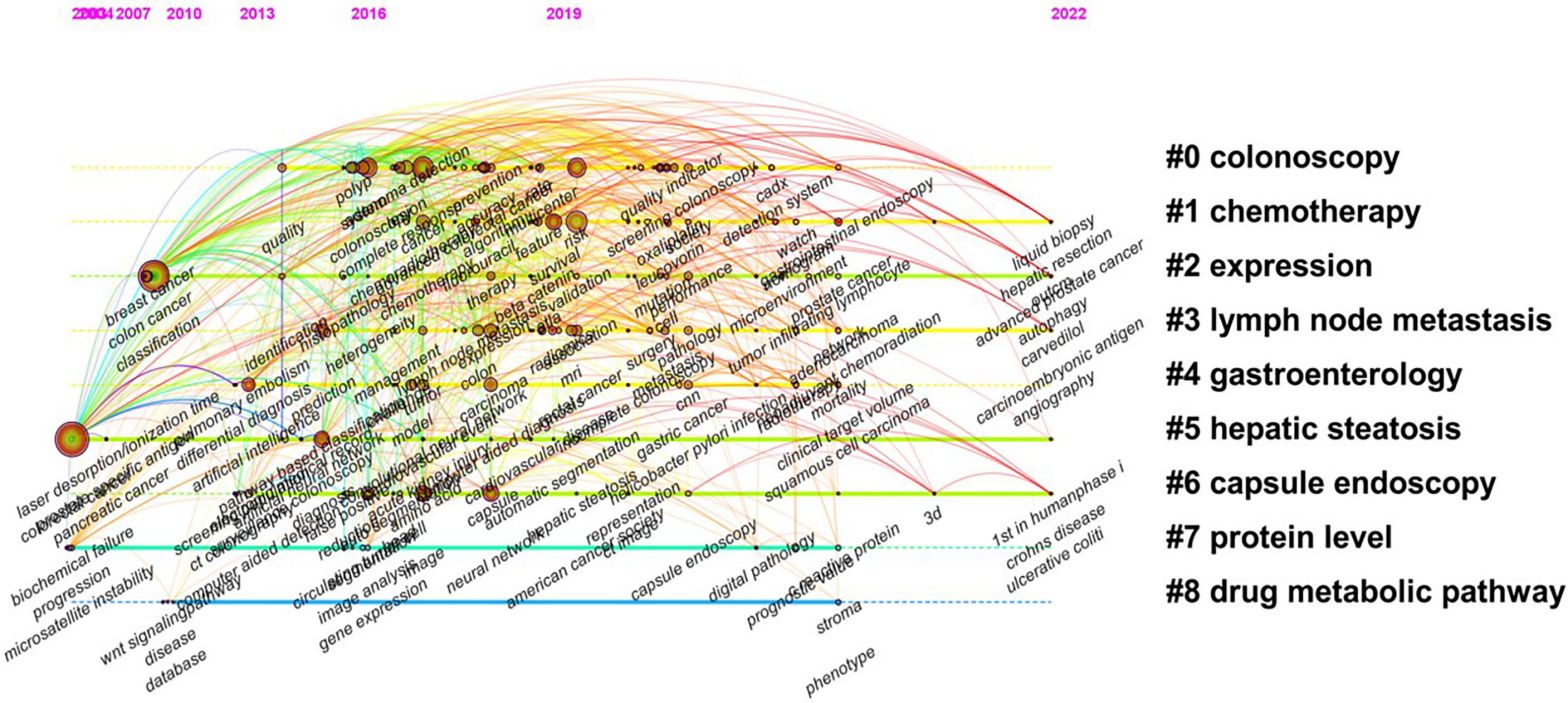
Co-citation timeline of references in studies on AI in colorectal cancer.

Using the Timeline view mode of the CiteSpace software, the variation in the frequency of literature citations was analyzed. **Figure 9** depicts a timeline view of the number of Chinese land contributions in each cluster. The greater the number of Chinese contributions in each cluster, the greater the significance of the cluster domain, reflecting the time characteristics of clustering. Analysis revealed that the clusters were representative of colonoscopy, chemotherapy, expression, lymph node metastasis, hepatic steatosis, and capsule endoscopy. Over time, it was possible to observe the flow of research topics between clusters. The literature about colonoscopy, chemotherapy, lymph node metastasis, and capsule endoscopy has been cited more frequently, which has made this field a hot topic. The reference lines between adjacent topics indicated the source and origin of research hotspots.

## Frontier dynamic analysis

**Figure 10:**
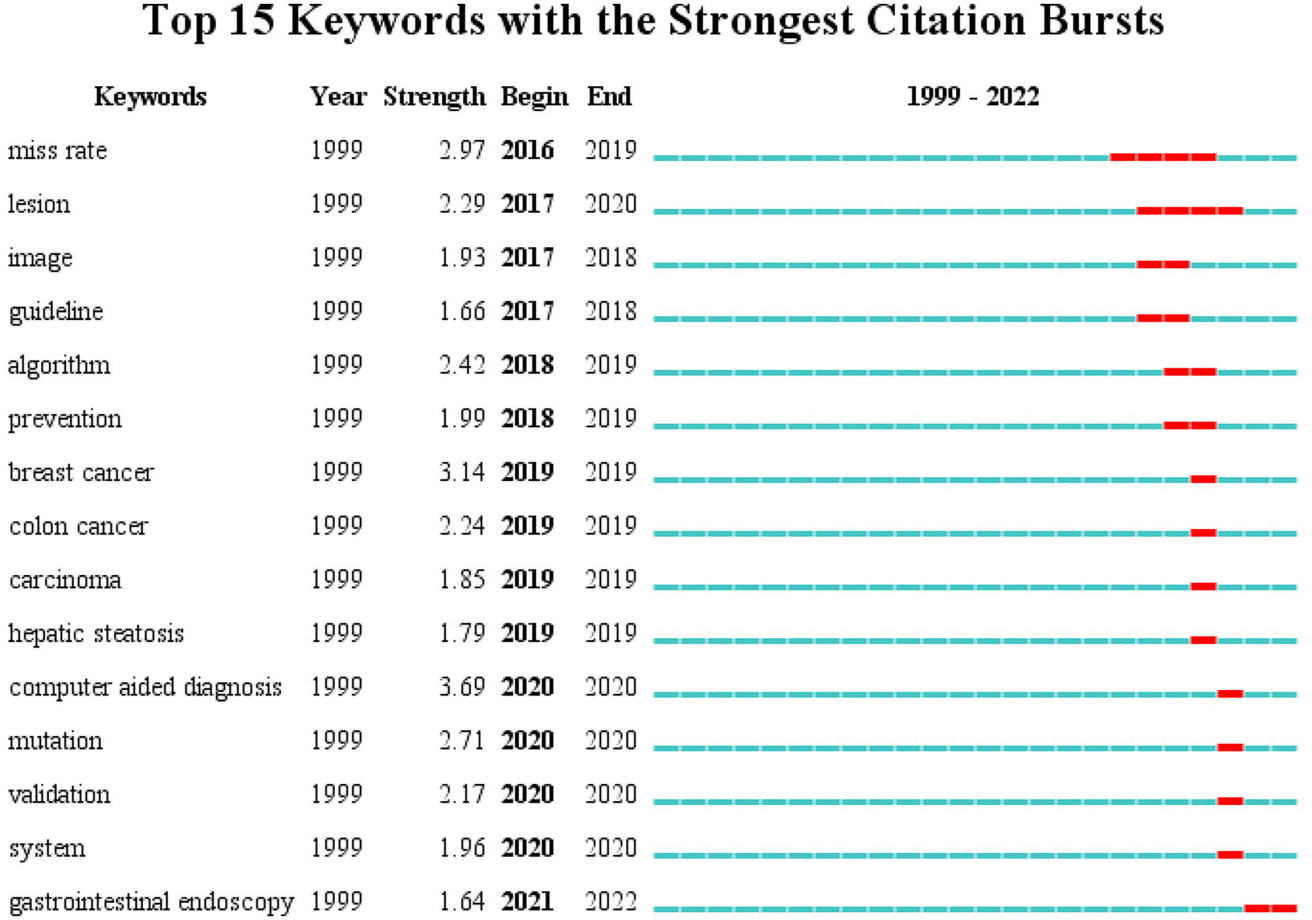
Burst map of kevwords.

Emergent words are keywords whose frequency of occurrence or usage increases dramatically over a short period. Emergent words can reflect the significant junctures of research hotspots during a given period. Based on an analysis of keyword cluster maps and with the assistance of CiteSpace’s emergent word detection function, the academic frontier in this field has been identified. **Figure 10** displays the top 15 emergent words, with the red bar on the right representing the duration of the hot spot. The results revealed that the literature in this study was most frequently observed between 2017 and 2020, and many emergent words appeared, indicating that the scope of AI research in CRC is rapidly expanding. The research appears to have developed rapidly during this period and received constant attention. In addition, it is noteworthy that many early emergent words have disappeared since 2021.

## Literature citation analysis

Since the publication of the first article in this field in 1999, the 562 articles included in this study have been cited 8,629 times on average, resulting in an h-index of 42 and a mean of 15.35 citations per article. The top 10 most-cited articles were chosen, and the results (**Table 5**) revealed that the highest impact factor of the ten articles was 23.059. The highest citation of a single article was 1,202, resulting in a total of 3,373 citations which accounted for 39.09% of the total citations, an average citation of 337.3, and an impact factor of 12.568. The information above reflects the academic influence of these documents and the current research trends in this field. The results of the analysis of the highly cited articles are presented in Table 5.

**Table 5:** Top 10 most cited articles.

Rank	Title	Paper type	First author	Journal	Conntry	IF	Total citations	Time
1	Convolutional Neural Networks for Medical Image Analysis: Full Training or Fine Tuning? (10)	Article	Nima Tajbakhsh	IEEE Transactions on Medical Imaging	USA	10.048	1202	2016
2	Locality Sensitive Deep Learning for Detection and Classification of Nuclei in Routine Colon Cancer Histology Images (11)	Article	Sirinukunwattana	IEEE Transactions on Medical Imaging	UK	10.048	515	2016
3	Deep Learning Localizes and Identifies Polyps in Real Time With 96% Accuracy in Screening Colonoscopy (12)	Article	Urban	Gastroenterology	USA	22.682	274	2018
4	Real-time automatic detection system increases colonoscopic polyp and adenoma detection rates: a prospective randomised controlled study (13)	Article	Pu Wang	Gut	China;USA	23.059	241	2019
5	Deep Learning in Label-free Cell Classification (14)	Article	Chen, Claire Lifan	Scientific Reports	USA	4.380	222	2016
6	Deep learning based tissue analysis predicts outcome in colorectal cancer (15)	Article	Bychkov, Dmitrii	Scientific Reports	Finland;Sweden;UK	4.380	221	2018
7	The Applications of Radiomics in Precision Diagnosis and Treatment of Oncology: Opportunities and Challenges (16)	Review	Liu, Zhenyu	THERANOSTICS	China	11.556	190	2019
8	Accurate Classification of Diminutive Colorectal Polyps Using Computer-Aided Analysis (17)	Article	Chen, Peng-Jen	Gastroenterology	Chinese Taipei	22.682	178	2018
9	Predicting survival from colorectal cancer histology slides using deep learning: A retrospective multicenter study (18)	Article	Kather, Jakob Nikolas	Plos medicine	Germany	11.069	170	2019
10	Automatic Detection and Classification of Colorectal Polyps by Transferring Low-Level CNN Features From Nonmedical Domain (19)	Article	Zhang, Ruikai	IEEE Journal of Biomedical and Health Informatics	Hong Kong SAR	5.772	160	2017

## Discussion

### Current research status and achievements

From 1999 to 2022, 562 research articles were published in this field by 300 authors, 230 research institutions, 245 journals, and 64 countries or regions. Since 2016, the number of published papers has increased exponentially in tandem with the continuous maturation of AI technology. China and the USA are the countries with the most active researchers in this field. They also demonstrate the closest cooperation, indicating that these two countries play significant roles in this field. The research conducted in the USA and the UK is generally of high quality, and the World Journal of Gastroenterology publishes the most articles in this field. In addition to fostering more significant research innovation and breakthroughs through collaborative efforts, close exchanges and cooperation among countries, research institutions, and academics can guide research to keep pace with the international research frontier and research hotspots.

### Research focus on AI in CRC

Cluster analysis illustrates the direction of research hotspots in this field, whereas the timeline view illustrates the evolution of relevant hotspots over time. For example, diagnosis and treatment, genes and immunology, intestinal polyps, tumor grading, and prognosis are research hotspots. When keyword co-occurrence is combined with highly cited papers, research hotspots and frontiers can be more accurately identified and detected.

Cluster analysis demonstrates that the researcher is most interested in diagnosis and treatment, with the algorithm being the most important keyword. *Evolutionary neural networks for medical image analysis: full training or fine tuning?*, published in IEEE Transactions on Medical Imaging in 2016 and authored by Nima Tajbakhsh from Arizona State University, was the most-cited article (1,202 times) (10). Based on four different medical imaging applications from three different imaging modality systems, the research demonstrated that deeply fine-tuned Convolutional Neural Networks (CNNs) are helpful for medical image analysis, outperforming fully trained CNNs when limited training data is available. This study’s related algorithm optimization research pertains to the clustering of diagnosis and treatment. Meanwhile, *Locality sensitive deep learning for detection and classic*, published in IEEE Transactions on Medical Imaging in 2016 and authored by Sirinukun Wattana from the University of Warwick, UK, ranked second with 515 citations (11). The research demonstrated neighborhood-aware deep learning approaches for nucleus detection and classification in routinely stained histology images of colorectal adenocarcinomas. The novel Neighboring Ensemble Predictor (NEP) and CNN combination could provide a systematic quantitative analysis of tissue morphology and tissue constituents, making it a valuable tool for a deeper understanding of the tumor microenvironment.

The prognosis of tumors is closely related to genetics and immunology, and AI technology has also produced fruitful results in this field. For example, Zhang et al. (20), used computer propagation artificial neural network (CP-ANN) and near-infrared spectroscopy to detect the mutation of BRAF gene V600E in CRC pathological specimens with a sensitivity of 93.8%. This study demonstrates that AI is reliable for detecting CRC gene mutations and has the benefits of simple, rapid, and inexpensive sample preparation.

Moreover, studies have demonstrated that CD3 and CD8 infiltration are strongly associated with the prognosis of CRC. The quantitative analysis of immune cells infiltrating the tumor performed better than tumor-intrinsic prognostic variables. According to related research, AI tools can detect additional prognostic markers on pathological sections. Reichling et al. (21), conducted a prospective study based on a pathological slide stained with CD3 and CD8 from 1,018 patients. They designed a new AI software (ColoClass) that utilized the random classification 32 machine learning model and the VSURF algorithm. To study the tumor-intrinsic prognostic variables CD3 and CD8 immune infiltration in stage III CRC, automated quantifying lymphocyte density and surface area in the tumor core and infiltrating margins (AUC=0.56) was performed. AI could assist pathologists in determining the prognosis for stage III colon cancer patients.

We must continue to pay attention to the issue of early CRC diagnosis, which is also a hotspot of current research. Neoplastic and non-neoplastic growths that protrude into the intestinal lumen are categorized as intestinal polyps. Adenomas are precancerous lesions that are easily transformed into CRC. The detection and removal of adenomas can prevent the development of CRC. Colonoscopy is primarily utilized for the differential diagnosis of polyps. It is anticipated that using AI technology during colonoscopy will improve colonoscopist performance, diagnostic accuracy, and polyp detection, classification, and isolation abilities. These modifications may result in increased adenoma detection rates and, ultimately, a reduction in CRC morbidity and mortality (22).

Four of the ten most-cited articles have significantly improved AI identification of intestinal polyps. First, Urban et al. (12), utilized CNN for computer-assisted image analysis to enhance the detection of polyps. The accuracy rate is 96.4%, and the area under the characteristic curve is 0.991. Second, Pu Wang et al. (13), confirmed that the real-time CADe system based on deep learning could significantly increase the detection rate of adenomas in individuals with a low prevalence of ADR (29.1% vs. 20.3%, p < 0.01), and the average number of adenomas per patient (0.53 vs. 0.31, p < 0.001), which is also the article with the highest impact factor. Third, Chen et al. (14), developed the DNN-CAD system to identify colorectal polyps smaller than 5 mm that are neoplastic or proliferative. This system classifies polyps with a PPV of 89.6% and NPV of 91.5%, and it is faster than endoscopy. Finally, Zhang et al. (19), developed a CNN algorithm to detect and classify hyperplastic and adenomatous colorectal polyps. The results indicate that the proposed method is as accurate as the visual inspection by endoscopists (87.3% vs. 86.4%), but the recall rate is higher (87.6% vs. 77.0%), and the accuracy is higher (85.9% vs. 74.3%). On the other hand, the high number of citations of these four articles confirms that the application of AI in gastrointestinal endoscopy has become a hot topic of research since 2021.

In addition to those above highly cited papers, Yamada M developed a real-time, robust AI diagnostic system for CRC that can significantly reduce the risk of missed diagnosis of nonpolyposis lesions during colonoscopy (23). The sensitivity and specificity of the AI system were 97.3% and 99.0%, respectively, with an AUC of 0.975. This AI system can remind endoscopists in real time to avoid missing the diagnosis of nonpolypoid polyps during colonoscopy. It is expected to compensate for the disparity in diagnostic quality between physicians of different levels and improve the early detection of CRC. In recent years, optical coherence tomography (OCT) has emerged as one of the most promising new tomographic techniques, particularly for tissue detection and imaging. Zeng et al. (24), developed a CNN pattern recognition optical coherence tomography (PR-OCT) system based on 26,000 CT images of colonic mucosa, which can accurately diagnose colon cancer mucosa in real-time with computer assistance. Sensitivity is 100%, specificity is 99.7%, and the area under the ROC curve (AUC) is 0.998. The system is anticipated to aid physicians in real-time screening and treatment evaluation of early mucosal tumors.

The colonoscopy diagnosis system based on AI technology has the advantages of reducing the missed diagnosis rate of CRC lesions, shortening the examination time, and bridging the diagnostic quality gap between different levels of endoscopists in comparison to traditional endoscopy. It is anticipated to be an invaluable resource for early cancer detection.

This study has some limitations. First, the WoSCC database is the only data source analyzed in this study. The VOSviewer software can only perform statistical analysis on the contents of a single database, which may produce biased results. Nevertheless, as one of the most extensive databases in the world, the WoSCC database chosen for this study is regarded as the best database for bibliometric analysis. Second, only the literature in the English language is included in this study; literature in other languages is excluded. China, the most active nation in this field, has also published many research results in Chinese. However, English is the language most commonly used for publishing academic articles, so the results of this study are still reliable. Due to the short publication time of recently published high-quality and ground-breaking achievements, the citation frequency of the primary assessment indicators of literature quality is low, which may affect the results of this study. On the other hand, bibliometric analysis allows us to determine AI’s current state and future development trends in CRC. The quantitative data index can provide global scholars with theoretical guidance for future research.

## Conclusion

This study used hybrid analysis and visualization techniques to examine the number of publications, countries, major research institutions, published journals, prominent authors, and their associated cooperation networks. These results can provide future researchers with guidance on potential opportunities for collaboration. Furthermore, through bibliometric analysis, this study also reveals objectively and exhaustively the current research hotspots and frontiers, thereby providing researchers with valuable guidance for choosing future research directions.

## Data availability statement

The original contributions presented in the study are included in the article/supplementary material. Further inquiries can be directed to the corresponding author.

## Author contributions

GL and YL conceived and designed the work. GZ and GT collected the data and performed the analysis. GL wrote the original draft. SL and YL reviewed and revised the manuscript. All authors contributed to the article and approved the submitted version.

## Funding

This work was supported by Shandong Natural Science Foundation of China (grant numbers: ZR2019PF017 and ZR2020MF155).

## Conflict of interest

The authors declare that the research was conducted in the absence of any commercial or financial relationships that could be construed as a potential conflict of interest.

## Publisher’s note

All claims expressed in this article are solely those of the authors and do not necessarily represent those of their affiliated organizations, or those of the publisher, the editors and the reviewers. Any product that may be evaluated in this article, or claim that may be made by its manufacturer, is not guaranteed or endorsed by the publisher.
